# Viral community composition of hypersaline lakes

**DOI:** 10.1093/ve/vead057

**Published:** 2023-08-30

**Authors:** Callum Le Lay, Joshua N Hamm, Timothy J Williams, Mang Shi, Ricardo Cavicchioli, Edward C Holmes

**Affiliations:** Sydney Institute for Infectious Diseases, School of Medical Sciences, The University of Sydney, Sydney, NSW 2006, Australia; School of Biotechnology and Biomolecular Sciences, University of New South Wales, Sydney, NSW 2052, Australia; State Key Laboratory for Biocontrol, School of Medicine, Shenzhen Campus of Sun Yat-sen University, Sun Yat-sen University, Shenzhen, China; School of Biotechnology and Biomolecular Sciences, University of New South Wales, Sydney, NSW 2052, Australia; Sydney Institute for Infectious Diseases, School of Medical Sciences, The University of Sydney, Sydney, NSW 2006, Australia; Department of Marine Microbiology and Biogeochemistry, Royal Netherlands Institute for Sea Research, P.O. Box 59, Den Burg NL-1790 AB, The Netherlands

**Keywords:** archaea, hypersaline, RNA virus, bacteriophage, detection, RNA-dependent RNA polymerase

## Abstract

Despite their widespread distribution and remarkable antiquity no RNA viruses definitively associated with the domain *Archaea* have been identified. In contrast, 17 families of DNA viruses are known to infect archaea. In an attempt to uncover more of the elusive archaeal virosphere, we investigated the metatranscriptomes of hypersaline lakes that are a rich source of archaea. We sequenced RNA extracted from water filter samples of Lake Tyrrell (Victoria, Australia) and cultures seeded from four lakes in Antarctica. To identify highly divergent viruses in these data, we employed a variety of search tools, including Hidden Markov models (HMMs) and position-specific scoring matrices (PSSMs). From this, we identified 12 highly divergent, RNA virus-like candidate sequences from the virus phyla *Artverviricota*, *Duplornaviricota*, *Kitrinoviricota, Negarnaviricota*, and *Pisuviricota*, including those with similarity to the RNA-dependent RNA polymerase (RdRp). An additional analysis with an artificial intelligence (AI)-based approach that utilises both sequence and structural information identified seven putative and highly divergent RdRp sequences of uncertain phylogenetic position. A sequence matching the *Pisuviricota* from Deep Lake in Antarctica had the strongest RNA virus signal. Analyses of the dinucleotide representation of the virus-like candidates in comparison to that of potential host species were in some cases compatible with an association to archaeal or bacterial hosts. Notably, however, the use of archaeal CRISPR spacers as a BLAST database failed to detect any RNA viruses. We also described DNA viruses from the families *Pleolipoviridae*, *Sphaerolipoviridae*, *Halspiviridae*, and the class *Caudoviricetes*. Although we were unable to provide definitive evidence the existence of an RNA virus of archaea in these hypersaline lakes, this study lays the foundations for further investigations of highly divergent RNA viruses in natural environments.

## Introduction

The *Archaea* (previously Archaebacteria) were originally distinguished from the *Bacteria* though the analysis of ribosomal RNA (rRNA) sequence data (Woese and Fox 1977). Following this landmark work, the sequencing and phylogenetic analysis of rRNA were widely employed to identify and describe entire microbial communities (Pace et al. 1986). These early investigations of the diversity of domains *Archaea* and *Bacteria* were followed by the development of metagenomics, in which the entire nucleic acid composition within a sample could be sequenced, with individual microbial genomes reconstructed using increasingly sophisticated bioinformatics pipelines. Whether based on total RNA or DNA sequencing, metagenomics has transformed microbiology, and greatly expanded the known diversity of bacteria, archaea, and eukaryotes (Hugenholtz and Tyson 2008), as well as of the viruses that infect them (Zhang, Shi, and Holmes 2018).

Despite the expansion of the viral universe through metagenomics, including the increasingly rapid discovery of viruses in diverse bacteria and eukaryotes, a notable absence is the definitive identification of RNA viruses in members of the domain *Archaea* (Bolduc et al. 2012; Wolf et al. 2020). There are three explanations for this apparent absence: that RNA viruses have never been associated with archaea, that RNA viruses do infect archaea but are rare, or that archaeal RNA viruses are so highly divergent in gene sequence that they are missed by metagenomics-based methods based on sequence similarity comparisons. Tangential evidence against the former hypothesis is that a wide variety of DNA viruses, which likely originated after RNA viruses according to the ‘RNA world’ hypothesis (Koonin, Senkevich, and Dolja 2006), are found in archaea, such that these organisms are accessible to at least some form of viral infection. Regardless of which theory prevails, identifying RNA viruses within archaea would be of enormous importance to our understanding of the origins and early evolution of viruses, and perhaps of all life (Holmes 2011; Krupovic, Dolja, and Koonin 2019).

Paradoxically, the lack of known archaea-infecting RNA viruses may in part reflect the growing reliance on metagenomics for virus discovery. While identifying novel viruses based on indices of sequence similarity is feasible and powerful when the target viruses are related to those viruses already present in sequence databases, it may fail when virus genomes are too distantly related to be utilised in meaningful comparisons. Indeed, given the rapidity of RNA virus evolution in which little or no sequence similarity is expected to remain after billions of years of evolutionary change (Holmes 2009), it is reasonable to expect that archaeal viruses will be highly divergent in gene sequence due to the phylogenetic distance of their hosts from the bacteria and eukaryotes typically sampled in studies of RNA virus diversity. For reference, in the case of the most conserved gene region in RNA viruses—the RNA-dependent RNA polymerase (RdRp)—the pairwise identity between supposedly aligned amino acid sequences is often less than the 5 per cent expected by chance alone, and can be as low as 1 per cent, in turn compromising phylogenetic analyses (Holmes, Duchêne, and Racaniello 2019). Sequence comparisons to any putative viruses of archaea may be even more challenging.

Accurately assigning virus–host relationships is another major challenge for metagenomics because nucleic acid sequences can be derived from multiple sources (i.e. the host in question or elements of its diet or microbiome). Indeed, it is difficult to formally prove that a virus infects a particular host using metagenomic data alone. In the case of the RNA viruses of archaea, Bolduc et al. (2012) identified an RNA virus sequence from an archaea-dominated microbiome that was initially proposed to be an archaea-associated virus. With the later expansion of RNA virus diversity following metagenomic studies, the host of this virus was revised to be an unknown eukaryote (Young et al. 2013; Wolf et al. 2020). Hence, detecting an RNA virus in an archaea-dominated community alone is insufficient evidence to prove that is a *bona fide* archaeal virus.

In contrast to RNA viruses, a wide variety of DNA viruses that infect archaea have been identified, which exhibit a diverse variety of capsid morphotypes: bottle-shaped (*Ampullaviridae*), spindle-shaped (*Bicuadaviridae*, *Fuselloviridae*, and *Salterprovirus*), coil-shaped (*Spiraviridae*), droplet-shaped (*Guttaviridae*), filamentous (*Rudiviridae, Lipothrixviridae*, *Clavaviridae,* and *Tristromaviridae*), pleomorphic morphology (*Pleolipoviridae*), and icosahedral with contractile tails (*Caudoviricetes*) (Snyder, Bolduc, and Young 2015; Krupovic et al. 2018; Dávila-Ramos et al. 2019). In archaea of the phyla *Halobacteriota* (i.e. the halophilic archaea, or ‘haloarchaea’) and *Nanohaloarchaeota* that inhabit hypersaline environments DNA viruses from the class *Caudoviricetes* and order *Ligamenvirales* have been documented. Importantly, DNA viruses are also key drivers of microbial evolution in archaea-dominated microbiomes (Held and Whitaker 2009; Snyder, Bolduc, and Young 2015), and such a diversity of capsid morphologies is testament to their antiquity and evolutionary flexibility.

Herein, we attempt to offset some of the drawbacks of metagenomic virus discovery by searching for divergent RNA viruses using methods that do not only rely on the sequence similarity of putative new viruses to already known viruses. First, we screened metagenomic data for the canonical RdRp motifs that are broadly conserved across all the RNA viruses described to date (Charon et al. 2022; Edgar et al. 2022). There are three RdRp motifs that are critical to the function of replicases, denoted the A, B, and C motifs. These can be detected using PSSMs as used by the Palmscan tool (Babaian & Edgar, 2022) or HMMs in the RdRp-scan protocol (Charon et al. 2022). The C motif, which encodes a loop containing a GDD amino acid triplet flanked by beta strands, is the most conserved as it is essential for the formation of a metal ion binding site in the RdRp needed for RNA strand elongation (Venkataraman, Prasad, and Selvarajan 2018). To supplement this analysis, we employed a recently developed AI approach (denoted ‘LucaProt’) that utilises both sequence and structural information and which has greatly expanded the known biodiversity of RNA viruses, including the discovery of multiple new clades of RNA bacteriophage (Hou et al. 2023).

Second, we searched for RNA viruses by matching candidate sequences to the spacers of the Clustered Regularly Interspaced Short Palindromic Repeat (CRISPR) regions of host taxa (Terns and Terns 2011). CRISPR/Cas systems are utilised by 80 per cent of archaea, with the implication that these are used to impair invading foreign nucleic acids (Luk et al. 2014). The CRISPR region itself comprises short regions of ‘non-self’ sequences, called spacers, that fall between short direct repeats. These spacer regions are complementary to foreign nucleic acids and are incorporated as a form of ‘immune memory’. The CRISPR/Cas system expresses and extracts these spacer regions, which are then employed to recognise invading nucleic acids. Crucially, because these spacers have sequence similarity to DNA viruses and can potentially have similarity to RNA viruses, they can also potentially help to identify divergent RNA viruses (Wolf et al. 2020). The benefits of this approach are that it does not require sequence similarity to a set of known viruses and that evidence for host attribution is acquired simultaneously.

Finally, it might also be expected that archaeal RNA viruses would have biases in dinucleotide composition that reflect those of their archaeal hosts. Dinucleotide frequencies are not randomly distributed products of nucleotide frequencies, with some dinucleotides over- or under-represented in specific organisms For example, eukaryotes suppress CpG while this dinucleotide is overrepresented in some α-proteobacterial and halobacterial sequences (Karlin, Mrázek, and Campbell 1997). Hence, it is possible to record the representation of all 16 dinucleotides to create a dinucleotide signature, which often reflects that of their broad host groups (Kapoor et al. 2010; Di Giallonardo et al. 2017). This representation can then be analysed by calculating the dinucleotide odds ratio: the frequency a dinucleotide is observed over its expected frequency given a random distribution.

At present, two families of RNA viruses are definitively known to infect bacterial species, although additional lineages have been identified (Hou et al. 2023). One of these, the *Fiersviridae* (previously *Leviviridae*), are positive-sense single-stranded RNA (+ssRNA) viruses that fall within the phylum *Lenarviricota* that contains the largely microbial fungus-infecting families *Narnaviridae* and *Mitoviridae* (Sadiq et al. 2022) (although the *Mitoviridae* specifically infect the mitochondria of fungi). The only other RNA virus family currently confirmed to infect bacteria are the *Cystoviridae* that have a double-stranded RNA (dsRNA) genomes. Given their basal phylogenetic position and simple structure, which only comprises a replicase protein in the case of the *Narnaviridae*, it is a reasonable hypothesis that archaea-associated RNA viruses could have sequences and structures similar to those seen in the *Lenarviricota*. In addition, the *Lenarviricota* are associated with an extensive range of bacteria, and because these bacteria also often inhabit the same habitats as archaea, they clearly have opportunities to co-exist and transmit to other hosts in archaeal habitats. If archaea-associated RNA viruses are indeed akin to the *Lenarviricota*, we might expect them to possess relatively small genome sizes, from approximately 2,500 to 7,000 nucleotides, and simple icosahedral capsids (Callanan et al. 2018), or no capsid at all as seen in the *Narnaviridae*.

The central aim of this study was to characterise the virome of archaea-dominated hypersaline lakes, particularly to assess whether the archaea present in these environments might also harbour RNA viruses. To do so, we obtained environmental samples from Lake Tyrrell in Victoria, Australia, for which 84 per cent of the microbial community comprises archaea (in particular *Haloquadratum walsbyi*; Podell et al. 2014), and is currently used by tourism and salt extraction companies. In addition, we examined archaeal enrichments from hypersaline Antarctic lakes collected in 2014. These microbial communities have generated studies on DNA viruses infecting haloarchaea of the family *Halobacteriaceae* (DeMaere et al. 2013; Tschitschko et al. 2015, 2018).

## Materials and methods

### Sample collection

Samples were collected from Lake Tyrrell, a hypersaline lake in Victoria, Australia (geographical co-ordinates −35.3230969, 142.79243448), between 20th and 23rd of August 2018, with the cooperation of the land managers, Cheetham Salt. Based on previous research (Podell et al. 2014), we believed this lake to be dominated by archaea. We collected 1 l samples from the top 30 cm of lake water, and accumulated biomass on 47 mm, 0.2 µm polyethersulphone membrane filters. These filters were stored immediately in 5 ml of RNAlater (Thermo Fisher, Waltham, USA) and kept at 4°C, then at −80°C on return to Sydney.

We were also able to produce archaea-rich samples by growing cultures seeded from four enrichments of water filter samples taken from east Antarctica: (i) Rauer 3 Lake (−68.814976, 77.827868) collected on Filla Island in September 2014, (ii) Rauer 6 Lake (−68.884850, 77.832541) on Torckler Island in September 2014, (iii) Club Lake, Vestfold Hills (−68.5417333, 78.2467667) in November 2014, and (iv) Deep Lake, Vestfold Hills (−68.556300, 78.187767) in November 2014. Enrichment cultures were produced from these samples and were stored at −80°C. We re-enriched these cultures in August 2019, using two types of media: modified David Burns cultivation media 2 (DBCM2) and artificial deep lake vitamin broth (ADLVB) (Erdmann et al. 2017; Hamm et al. 2019). Cultures were incubated at room temperature for 4–6 h, and then biomass was accumulated on 0.2 µm polyethersulphone membrane filters before immediate extraction of RNA that was stored at −80°C. The lake, media, and resulting sequencing library identifiers are described in Table 1.

**Table 1.** The hypersaline lakes sampled in this study, the media used for culturing of the microbial communities (if any), and the identifiers for the sequencing libraries generated.

Library ID	Enrichment media 1	Enrichment media 2	Lake sampled
LT3	–	–	Lake Tyrrell, Australia
LT9	–	–	Lake Tyrrell, Australia
CAA	ADLVB	ADLVB	Club Lake, Antarctica
CAD	ADLVB	DBCM2	Club Lake, Antarctica
DAA	ADLVB	ADLVB	Deep Lake, Antarctica
DAD	ADLVB	DBCM2	Deep Lake, Antarctica
DDA	DBCM2	ADLVB	Deep Lake, Antarctica
FA	ADLVB	–	Rauer 3 Lake, Filla island, Antarctica
FD	DBCM2	–	Rauer 3 Lake, Filla island, Antarctica
TA	ADLVB	–	Rauer 6 Lake, Torckler island, Antarctica

### Extraction, library preparation, and sequencing

RNA was extracted from samples using the RNeasy PowerWater Kit (QIAGEN, Hilden, Germany), and quantified using a 2200 Tapestation system (Agilent, Santa Clara, USA). Ribosomal RNA (rRNA) was not depleted as no effective means to remove archaeal rRNA was available. After isolation of RNA, read libraries were prepared, using a TruSeq Stranded mRNA Total Library Prep kit, and sequenced by the Australian Genome Research Facility using the NovaSeq 6000 platform (100bp paired-end; Illumina, San Diego, USA). Sequence reads were prepared using FastQC (v0.11.8; Andrews 2010) and Trimmomatic (v0.38; Bolger, Lohse, and Usadel 2014), and rRNA reads were removed computationally after identification using SortMeRNA (v2.1b); Kopylova, Noé, and Touzet 2012). Finally, all contigs were assembled using Trinity (v2.5.1; Grabherr et al. 2011), with Bowtie2 (v2.2.5; Langmead and Salzberg 2012) used to map reads back to contigs.

### Initial sequence similarity searches

**Figure 1.**
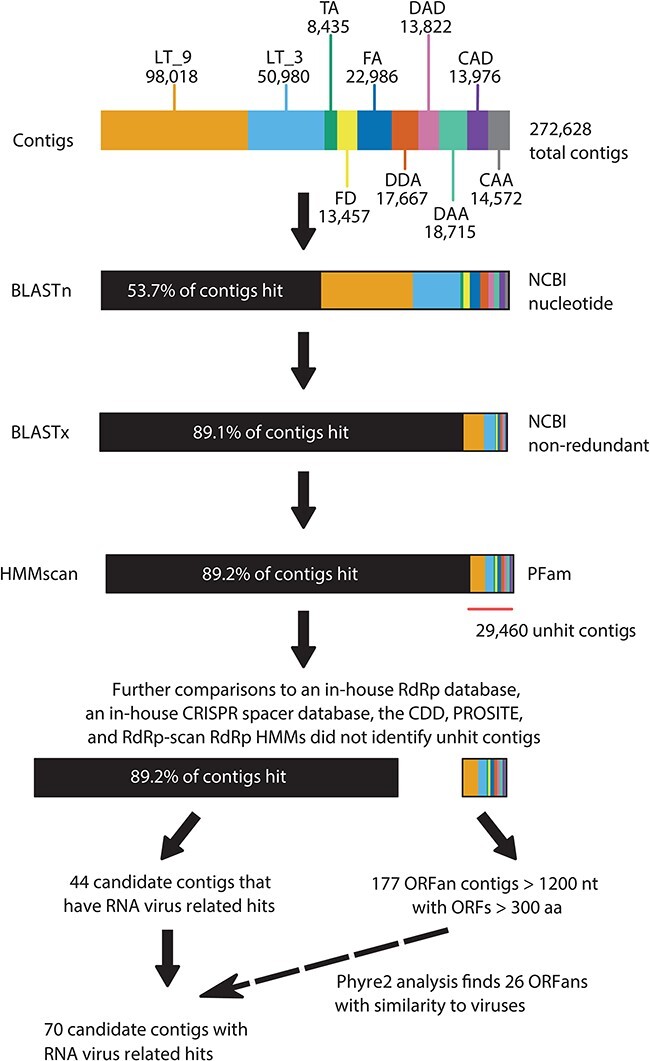
The analytical approach used to identify contigs obtained from the hypersaline lake sequencing libraries, with descriptions of the total number of contigs identified at key steps. The top bar represents the contigs analysed in this study, coloured by read library from which they were assembled. The bars following this colour depict the result of the specific search tool (and database compared) as applied the contigs, with contigs with a significant hit from that particular tool/database coloured black.

The overall analytical (bioinformatics) approach to identify candidate sequences is shown in Fig. 1. Contigs were identified using BLAST (BLAST+; v2.6.0, Camacho et al. 2009). Initially, Diamond BLAST (v0.9.25; Buchfink, Xie, and Huson 2015) was used to estimate the representation of viral and archaeal species in the sequencing libraries. Following assembly, we performed BLASTx searches of contigs to the NCBI non-redundant database and to an in-house curated data base of virus RdRps. Contigs that remained unidentified (i.e. that exhibited no significant matches) were collated and searched again using the (i) Reverse Position-Specific BLAST (RPS-BLAST; Marchler-Bauer et al. 2002) to the Conserved Domain Database (CDD; Lu et al. 2020) and (ii) HMMscan (HMMer; v3.3; Mistry et al., 2013) to the Pfam and PROSITE databases (Sigrist et al. 2012). The hits obtained for each analysis are described in Supplementary Table 1.

Following these second-round searches, any remaining unidentified contigs that were >1,200 nt in length and had a >300 amino acid open reading frame (ORF) (arbitrary cut-offs for the purposes of a manageable analysis) were analysed using the Phyre2 server (v2.0) that examines homology between amino acid sequence queries and records in the Protein Data Bank (PDB) to build models of protein structure (Kelley et al. 2015). Because of computational constraints, we performed this search with a subset of the sequencing library that contained plausible viral contigs, rather than searching the full assembly—that is, contigs that were >1,200 nt in length, had a >300 amino acid ORF, and had a hit to a viral sequence from a previous search. The EMBOSS package (v6.6.0; Rice, Longden, and Bleasby 2000) tool getORF was used to predict ORFs.

We also attempted to detect the sequences of highly divergent RNA viruses using the CRISPR spacer regions of archaea. These regions comprise repeating motifs interspaced with short (∼12nt) sequences (spacers) that are complementary to virus genomes. We used Entrez Direct (Kans 2013) to download the CRISPR region ‘cassettes’ that were annotated in the genomes of 1,827 archaea species from the NCBI nucleotide database. We then extracted the spacers from these cassettes by using regular expressions to detect and remove the repeating motifs in python (v3.10), leaving the spacers. Spacers that had significant BLAST hits (*P* < 0.005) to non-RNA-virus sequences were not included in further analysis. We then created a BLAST database from these curated spacers, to which we queried our assembled contigs.

### Detection of divergent RdRp sequences

RNA viruses necessarily carry a replicase gene, either an RdRp or a reverse-transcriptase (RT), such that detecting these sequences is commonly used to identify RNA virus sequences in metagenomic data. To detect the canonical A, B, and C motifs within the viral RdRps, as well as sequence motifs associated with RTs, all sequence assemblies were searched using Palmscan (Edgar et al. 2022) which compares sequences to PSSMs. This program was initially run with the default -hiconf flag, such that only high confidence hits were reported. After no credible hits were obtained, the Palmscan analysis was repeated with the -lowconf flag. In this mode, PalmScan allows for hits where there is low confidence in the C motif or where the three RdRp motifs are arranged in a non-canonical order. As it is the most conserved, the C motif is the most important for the reliable detection of RdRps.

As an additional method to detect highly divergent RNA viruses through the presence of RdRp signatures, we employed the recently developed RdRp-scan bioinformatic toolkit (Charon et al. 2022). This protocol provides phylogenetic trees, core sequences, motifs, and HMMs from the large-scale collection and alignment of RdRp sequences obtained from NCBI databases. We used the HMMs from this tool with HMMscan to search all assemblies for divergent RNA virus RdRp sequences, with default settings and an *e*-value cutoff of 5e−5. RdRp motif sequences for virus-like candidates are shown in Supplementary Table 2.

### Selection and annotation of virus-like candidate sequences

To better annotate putative RNA virus sequences, we collated 70 contigs with virus-related hits to the nucleotide (NCBI), protein (translated, NCBI), in-house RdRp, PROSITE, PHYRE2, RdRpscan profile, or Palmscan profile databases (see Results). Importantly, we examined contigs with any virus-related hit, including those that were not the top hit or considered significant using typical score thresholds, thereby increasing the scope of our investigation to detect extremely divergent RNA virus genomes. However, this procedure necessarily also increases the number of false-positive results. To compensate for this, we scored contigs by the quality and quantity of both their best search tool hits and any virus-related hits. The strength of a putative virus hit is scored individually, then summed across the different research tools (and databases) to obtain an overall score for the whole contig. Hits that are scored highly are those that match viruses, are of high confidence, and are the best hits from their respective search tool/database. Our simple scoring procedure is as follows:


$${S_i} = \mathop \sum \limits_{j = 1} {A_{ij}}/\left( {{B_{ij}}*{C_{ij}}} \right)$$

For each contig *i* an *S_i_* score was calculated, representing the combined confidence score for the *i*th contig being a sequence from an RNA virus. This score is determined by each search tool hit *j* for contig *i*, with hits scoring higher where they are the best hit returned from the tool and show high confidence similarity to an RNA virus. Variable *A_ij_* is scored 1 where hit *j* is to an RNA virus, 0.5 for a DNA virus, and −1 for a non-viral sequence. Variable *B_ij_* is scored 1 where hit *j* is the top hit of the hits provided by its respective search tool and 4 where it is not (this ensures that hits that are not the best recovered from a search tool are scored low). Variable *C_ij_* is scored 1 where hit *j* is a high confidence hit and 2 where it is not. Hits are considered high confidence when the given *e*-value is >0.05, or greater than 70 per cent confidence for Phyre2 hits.

### Detection of RdRp-like sequence using LucaProt

All contigs were submitted to LucaProt, which detects RdRp sequences using both sequence and structural information through an AI (i.e. machine-learning) approach (Hou et al. 2023). For all the putative RdRp sequences identified by LucaProt, we attempted to assign them to one of 180 superclades (viral phyla/classes) of RNA viruses described by Hou et al. (2023). A detailed description of the LucaProt methodology and the RNA virus superclades is provided by Hou et al. (2023).

### Dinucleotide analyses

Profiles of dinucleotide odds ratios were calculated using the seqinr library in R (Charif and Lobry 2007), with ggplot2 used as a visualisation tool (Wickham 2016). Odds ratios were calculated using the relation *Pxy* = *Fxy*/*FxFy*, where *F* is the frequency of nucleotide *x*, *y*, or the dinucleotide *xy* (Di Giallonardo et al. 2017). Profiles were created for all contigs and for reference genomes from archaea (the species *Hqr. walsbyi* and *Halohasta litchfieldiae*), bacteria (*Salinibacter ruber*), and eukaryotes (*Dunaliella salina* and *Homo sapiens*), which we then compared to the dinucleotide profiles of the putative virus contigs. We expect the profiles of viral sequences to be similar to those of their true host taxa and we used this property to make inferences on the most likely host group (i.e. determining which host reference sequence had a dinucleotide profile most similar to those of the virus contigs). Principal components analysis (PCA) was used to create an ordination of the dinucleotide odds ratios to visualise the variation in profiles.

### DNA virus detection and phylogenetic analysis

We filtered BLASTx contig search results, collating the top three hits for each contig, to obtain sequence hits related to DNA viruses. These were then extracted, and the contigs sorted by type of virus protein (capsid, DNA polymerase, terminase, etc.) and by taxonomic group (at the level of virus family or genus). In addition, RPS-BLAST searches of these contigs were run to assist in inference of protein types, as many predicted proteins from archaeal DNA viruses are labelled as ‘hypothetical’.

We aligned the predicted amino acid sequences (aa) from these contigs with virus type species selected from the International Committee for Taxonomy of Viruses (ICTV) reports for their respective taxonomic groups. We used the L-INS-i algorithm in MAFFT (v7.45; Katoh and Standley 2013) to construct multiple sequence alignments of these sequences, which were then trimmed manually to remove gapped regions. All alignments were >300 aa residues in length. Phylogenetic trees of these data were then estimated using the maximum likelihood approach available in IQ-TREE (v1.6.12; Nguyen et al. 2015), employing the LG + F + Γ_4_ amino acid substitution model chosen according to Bayesian information criterion, and with ultrafast bootstrap and approximate likelihood-ratio test (aLRT) used to measure clade confidence.

### Description of microbial species in sequencing libraries

We used CCMetagen (v1.1.3; Marcelino et al. 2020) to classify contigs to their (non-viral) microbial species (i.e. archaea, bacteria, and eukaryotes) by mapping reads using KMA (v1.2.4; Clausen, Aarestrup, and Lund 2018) to the ‘ncbi_nt_no_env_11jun2019’ database provided by the CCMetagen. The output data were represented using figures produced using R, as described below.

### Statistical analysis and visualisation

The R packages ggplot2, ggtree, ape (Paradis, Schliep, and Schwartz 2019), and viridis (Garnier et al. 2021) were used to create figures. Inkscape was used for graphic manipulation.

## Results

We assembled a total of 126 million sequence reads into 272,628 contigs from 10 samples representing five hypersaline lakes: Lake Tyrrell (Victoria, Australia), Club Lake, Deep Lake, and two lakes in the Rauer Islands (Antarctica). Of these, the Australian samples were direct environmental samples, while those from Antarctic lakes were archaeal enrichments. The Antarctic lake samples were cultured in 2014 then stored at −80°C, before being re-enriched in 2019. After RNA extraction, we obtained 22 million reads that assembled into 148,998 contigs from the Lake Tyrrell samples (contig length in nucleotides: min = 201, max = 20,282, mean = 520), and a total of 104 million reads that assembled into 123,630 contigs from the Antarctic lake enrichments (contig length in nucleotides: min = 201, max = 29,463, mean = 1,133 nt).

**Figure 2.**
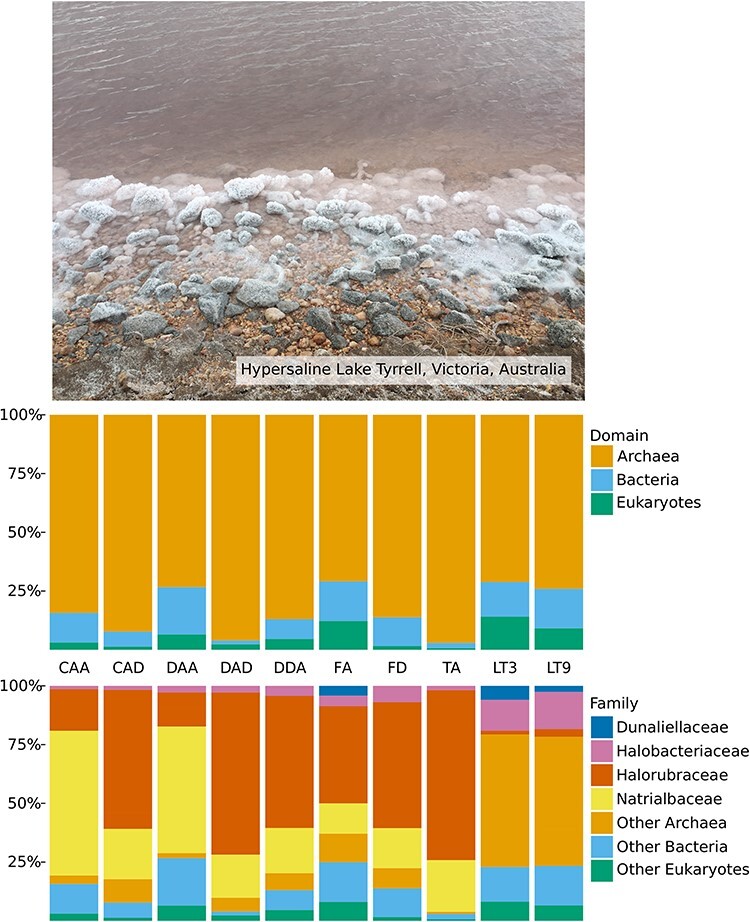
Relative abundance of (non-virus) microbes in all sequencing libraries, calculated using CCMetagen. The top panel displays a photo of Lake Tyrrell (Victoria, Australia) at the time of sampling on the 21st of August 2018. Library identifiers are given on the *x*-axis. The top graph depicts the relative abundance of microbes from the domains *Archaea, Bacteria*, and *Eukaryota*. The bottom graph shows the relative abundances of the families Dunaliellaceae (*Eukarya*), *Halobacteriaceae* (*Archaea*), *Halorubraceae* (*Archaea*), and *Natrialbaceae* (*Archaea*).

The samples utilised here were chosen for their archaea-dominated microbial communities which simplifies the attribution of RNA viruses to an archaeal host. To confirm this, we estimated host profiles for each library using CCMetagen (Fig. 2). For the Antarctic lakes, an average of ∼86 per cent of the sequence reads identified in these libraries were from archaea, with the families *Halorubraceae*, particularly *Halorubrum lacusprofundi*, and *Natrialbaceae*, particularly *Haloterrigena* sp. BsC5, representing the major archaeal components. As well as the archaea, we found on average 4 per cent eukaryotes and 10 per cent bacteria from the phyla *Actinomycetota* and *Proteobacteria*. For the Lake Tyrrell libraries, the microbial community profiles were on average 73 per cent archaea, particularly from the order Halobacteriales (44 per cent). There were also an average of 16 per cent bacteria, primarily of the genus *Salinibacter* and family *Ectothiorhodospiraceae*, and 12 per cent eukaryotes of the species *Dunaliella tertiolecta* and *Cladosporium herbarum*.

### Identification of putative RNA viruses

After confirming that our libraries represent archaea-dominated microbiomes, we searched for RNA virus sequences, particularly those that may have archaeal hosts. To do this, we attempted to identify all our contigs and in so doing separate host sequences from those that are likely to be viral (Fig. 1). Using this approach, we found significant hits (host or virus) for 243,184 contigs (89 per cent) using BLASTn to the NCBI nucleotide database, BLASTx to the NCBI non-redundant database, BLASTx to an in-house RdRp database, RPS-BLAST to the CDD, HMMscan to the PFAM database, HMMscan to the PROSITE database, Palmscan (in low confidence mode), and HMMscan to the RdRp-scan RdRp HMM database. In addition, we performed a BLASTn search with an in-house CRISPR spacer database, prepared from spacer regions obtained from archaeal genomes on NCBI.

Strikingly, these analyses returned no significant (*P* < 5e−5) hits to RNA viruses. They did, however, identify 44 contigs that had some similarity to virus genomes, which from here onwards are referred to as virus-like ‘candidates’. We also compiled a list of 177 contigs with no significant matches to any known protein, yet which had a length of >1,000 nt and an >300 aa ORF, suggestive of a viable protein; these are referred to as ‘ORFans’. We compared both candidate and ORFans contigs to available protein structures using Phyre2. Through this, we found that 26 of the ORFans from our candidate list were structurally similar to virus proteins. To increase sensitivity, contigs were considered candidates even with low confidence values or high *e*-values for their virus similarity.

Combined, these analytical steps identified 70 virus-like candidates: 44 virus-like candidates and 26 ORFans. However, our low threshold to denote ‘significance’ necessarily meant that the false-positive rate will also have increased. To address this issue, we created a combined confidence score (*S_i_*) for each contig (see Methods), allowing for low confidence results to collectively support identification of RNA viruses. This score summarises the results from each search tool/database described above: both the most significant result and any virus-related results for a candidate contig. Hence, the *S_i_* value for a contig is the sum of the scores calculated for each of its search tool results. A virus candidate is scored positively if it is linked to an RNA virus, less so if this link is to a DNA virus, and negatively if it links to a non-viral sequence.

**Figure 3.**
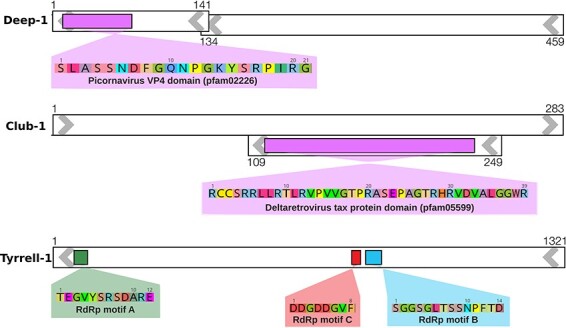
Genome schematics for the top-ranked candidate sequences: Deep-1, Club-1, and Tyrrell-1. White boxes represent open reading frames (ORFs), with arrows indicating orientation and the adjacent numbering showing the start and end ORF nucleotides. Coloured boxes indicate motifs detected by the search tools employed, and the translated amino acid sequences of these motifs are shown below in equivalently coloured magnified boxes.

Following this scoring exercise, 19 candidates were found to have a positive *S_i_*, meaning that they represent contigs containing virus-like sequences with potential to be *bona fide* viruses (Table 2). These contigs are <2,000 nt in length and at low abundance (<300 mapping reads), and each has only a single virus-related result from the most consecutive searches that are designed to detect highly divergent viruses: HMMscan to the PFAM database, Phyre2, and Palmscan. Ordered by *S_i_*, the first 12 of these 19 candidates were associated with RNA viruses and can be broadly grouped by phylum. Five candidate virus-like contigs were associated with the phylum *Kitrinoviricota* that comprise +ssRNA viruses and usually associated with eukaryotes. Three candidate contigs fall into the phylum *Pisuviricota*, a large and highly diverse group of +ssRNA viruses usually associated with a range of eukaryotes from fungi to vertebrates and plants. We also identified two candidates related to the phylum *Negarnaviricota* of -ssRNA viruses associated with eukaryotes. Finally, one candidate contig was related to each of the phyla *Duplornaviricota*—dsRNA viruses with members that infect both eukaryotes and bacteria—and the *Artverviricota*, retroviruses that typically infect eukaryotes. The putative amino acid motifs associated with the top three virus hits—denoted Deep-1 (*Pisuviricota*), Club-1 (*Artverviricota*), and Tyrrell-1 (*Kitrinoviricota*)—are shown in Fig. 3. Of note, Club-1 matched part of the Tax protein from the genus *Deltaretrovirus*, although the very short sequence meant that it was impossible to determine whether this was an endogenous or exogenous retrovirus sequence.

**Table 2.** The top 19 sequence candidates for virus genomes (12 RNA and 7 DNA) collated from the BLASTx, HMMscan, Palmscan (low confidence mode), and Phyre2 hits ordered by the degree of supporting evidence. The ‘Virus hit’ column gives the proteins matched in each case, with information on the database used, comments from the relevant search tool (e.g. motifs out of order), accessions, and confidence scores/*e*-values shown in parentheses.

Library	Candidate ID	Length (nt)	Reads	Detection method	Virus hit (comment)	Combined confidence score (Si)
*Candidates with RNA virus-like sequence*						
DDA	Deep-1	459	27	PFAM	*Pisuviricota (Picornavirus)* coat protein VP4 (PFAM: PF02226, e = 0.022)	1
CAA	Club-1	283	6	PFAM	*Artverviricota (Deltaretrovirus)* Tax protein (PFAM: PF05599, e = 0.016)	1
LT9	Tyrrell-1	1,321	37	Palmscan	*Kitrinoviricota* RdRP (motifs out of order)	0.5
LT9	Tyrrell-2	1,320	22	Palmscan	*Kitrinoviricota* RdRP (penalty low PSSM score, bad segment length)	0.5
LT3	Tyrrell-3	1,469	41	Palmscan	*Pisuviricota* RdRP (bad segment length)	0.5
LT9	Tyrrell-4	1,433	97	Palmscan	*Pisuviricota* RdRP (motifs out of order)	0.5
LT9	Tyrrell-5	1,264	46	Palmscan	*Duplornaviricota* RdRP (motifs out of order)	0.5
LT9	Tyrrell-6	1,306	35	Palmscan	*Kitrinoviricota* RdRP (motifs out of order)	0.5
DDA	Deep-2	1,495	148	Palmscan	*Kitrinoviricota* RdRP (penalty low PSSM score, bad segment length)	0.5
DAD	Deep-3	1,259	94	Palmscan	*Kitrinoviricota* RdRP (reject low C score)	0.5
CAA	Club-2	270	15	PFAM	*Mononegavirales* RdRp (PFAM: PF00946, e = 0.065)	0.5
CAA	Club-3	229	12	PFAM	Zinc-binding domain of *Paramyxoviridae* V protein (PFAM: PF13008, e = 0.068)	0.5
*Candidates with DNA virus-like sequence*						
LT9	Tyrrell-7	1,500	106	Phyre2	Oxidoreductase (PDB: 2 J5W, confidence = 45); bacteriophage T4 gp11 (PDB: 1EL6 chain A, Phyre2 confidence = 41.7)	0.38
LT9	Tyrrell-8	1,212	139	Phyre2	Canine adenovirus fibre head (PDB: 2J2J chain A, confidence = 31.2)	0.25
LT9	Tyrrell-9	230	10	PFAM	Curtovirus V3 protein (PDB: PF07436, e = 0.18)	0.25
LT9	Tyrrell-10	1,523	259	Phyre2	Head domain of porcine adenovirus type 4 (PDB: 2WST chain E, Phyre2 confidence = 43.6)	0.25
LT9	Tyrrell-11	1,879	76	Phyre2	Tailspike of *Acinetobacter baumannii* bacteriophage (PDB: 4Y9V chain A, Phyre2 confidence = 52.8)	0.06
LT9	Tyrrell-12	1,241	86	Phyre2	*Acidianus* filamentous virus 1 envelope (PDB: 5 W7G chain V, confidence = 48)	0.06
LT9	Tyrrell-13	1,209	98	Phyre2	Fowl aviadenovirus 1 major coat protein (PDB: 2INY chain A, Phyre2 confidence = 56.9)	0.06

Of the 12 RNA virus-like candidates, nine exhibited similarities to viral RdRps. The virus-like candidates most plausibly associated with the archaea were from the *Pisuviricota*, which are diverse and known to inhabit microbial environments, and the *Duplornaviricota* that are also known to infect microbial species. In contrast, the other phyla are usually associated with eukaryotes and hence perhaps less likely to be RNA viruses associated with archaea. However, all these putative sequences are so divergent that they could not be included in a viable phylogenetic analysis. Despite our hypothesis that the *Lenarviricota* was the viral phylum most likely to be associated with archaea, none of our top 12 RNA virus-like candidates were associated with this group.

The final seven virus-like candidates were from the Lake Tyrrell libraries and have the closest hits to DNA viruses: *Acinetobacter baumannii* bacteriophage, bacteriophage T4, canine adenovirus, porcine adenovirus, fowl aviadenovirus 1, curtovirus (family *Geminiviridae*), and acidianus filamentous virus 1 (family *Lipothrixviridae*). The protein database identifiers for these viruses are provided in Table 2.

We similarly applied the LucaProt methodology (Hou et al. 2023) to all the contigs obtained in this study. This revealed seven contigs that may represent an RdRp sequence: DN36333_c2_g3_i6 (981 nt) and DN36333_c2_g3_i8 (1032 nt) from library LT_9 (SRA accession SRR24125903), DN3293_c1_g9_i2 (6883 nt) and DN3363_c2_g3_i1 (3221 nt) from library CAD (accession SRR23196178), DN2424_c1_g1_i1 (2685 nt) and DN2424_c1_g1_i3 (2675 nt) from library CAA (accession SRR23196177), and DN2867_c1_g3_i1 (6871 nt) from library DDA (accession SRR23196181). Notably, however, none of these contigs could be placed into the superclade clusters described by Hou et al. (2023) such that their validity as *bona fide* RNA viruses and phylogenetic position requires additional verification. In addition, none of the nine RNA virus-like candidates with RdRp matches identified by the procedure described above (Table 2) were detected by LucaProt.

**Figure 4.**
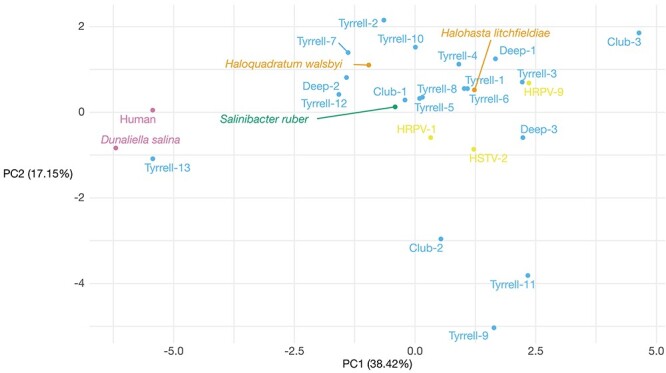
Scatter plot showing the first two principal components from a PCA of dinucleotide odds ratio profiles calculated for the top candidates (12 RNA virus-like candidates), as well as for reference sequences. Virus-like candidates are coloured in blue, bacteria are coloured green, archaea are coloured in orange, eukaryotes are coloured in pink, and DNA viruses of haloarchaea are coloured in yellow.

Finally, we calculated the dinucleotide odds ratios of our positive *S_i_* candidates, as well as for eukaryote, bacteria, archaea, and DNA virus reference genomes. The resulting PCA revealed that the first and second principal components explained 56 per cent of the variation in dinucleotide representation. Figure 4 represents all contigs with a point, with the distances measuring the similarity between their respective dinucleotide odds ratio profiles. This revealed that 10 of our RNA virus-like candidates (14 virus-like candidates in total) had broadly similar dinucleotide compositions to the archaeal species *Hqr. walsbyi* and *Hht.a litchfieldiae*, and to the bacterial species *S. ruber*, including those with the highest *S_i_* values. Hence, these candidates could be host-derived sequences—although this is unlikely given their hits to RNA viruses—or from RNA viruses infecting these archaeal or bacterial hosts. In particular, our most likely virus-like candidate, Deep-1, has a similar dinucleotide representation to archaea and bacteria with an over-representation of CpG and under-representation of CpA, although the sequence is only 459 nt in length. Interestingly, the dinucleotide representation of Deep-1 shows greater similarity to *Hht. litchfieldiae* than to *S. ruber* or *Hqr. walsbyi*. Outside this main cluster, we identified five candidates that fell into three distinct groups. Tyrrell-13 groups with the eukaryotic dinucleotide profiles from human and *D. salina* that have a characteristic over-representation of CpA and UpG and under-representation of CpG, suggesting that it was also derived from a eukaryotic host (Di Giallonardo et al. 2017). In contrast, Club-2, Tyrrell-9, and Tyrrell-11 are distinct from all other profiles due to an over-representation of ApA and under-representation of ApU and UpA, while Club-3 has an over-representation of UpA that differentiates it from the majority. The provenance of these sequences remains uncertain.

### Identification of DNA viruses

**Figure 5.**
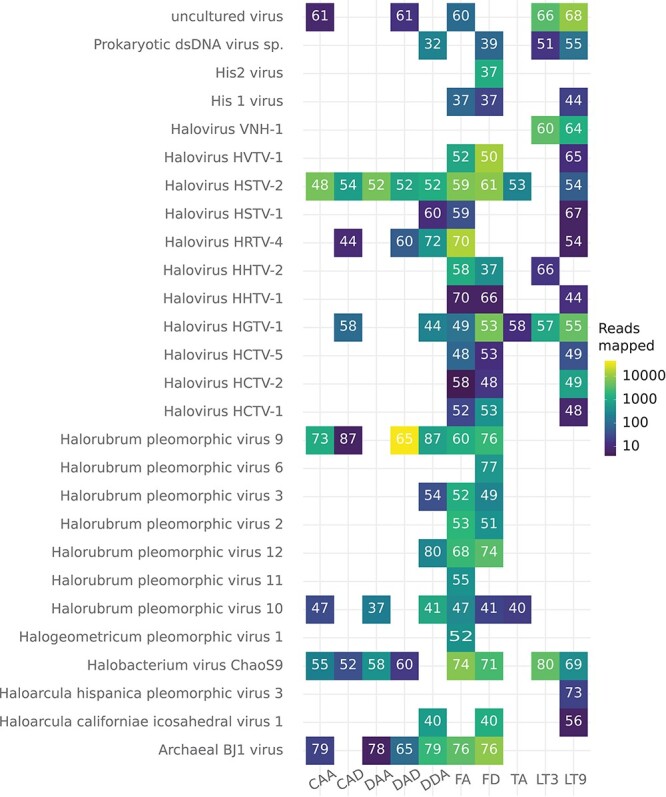
Heatmap of reads mapping to DNA virus-associated contigs detected using BLASTx. Each cell represents the presence of a DNA virus, given on the y-axis, in a library (Table 1) specified on the *x*-axis, and is coloured by the number of reads mapping. Read mapping is shown on a logarithmic scale, in which yellow cells have the most mapped reads, while dark blue cells have the least. Each cell contains the average percentage amino acid identity of the contigs to the DNA virus specified on the *y*-axis, as determined in the BLASTx search.

In addition to the seven putative DNA viruses identified above, initial BLASTx searches of our microbial communities identified contigs with an average of 61 per cent amino acid identity to haloarchaea-infecting DNA viruses. A total of 419 contigs from the Lake Tyrrell libraries matched 17 DNA virus species, 12 of which were tailed haloviruses from the class *Caudoviricetes*. Similarly, 423 contigs matched 25 DNA virus species identified in the Antarctic lake libraries, primarily the Filla island libraries FA (169 contigs) and FD (306 contigs), with 12 of these species falling as members of the *Caudoviricetes*. In total, 842 contigs matched viruses from the families *Pleolipoviridae*, *Sphaerolipoviridae*, and *Halspiviridae* (Fig. 5).

**Figure 6.**
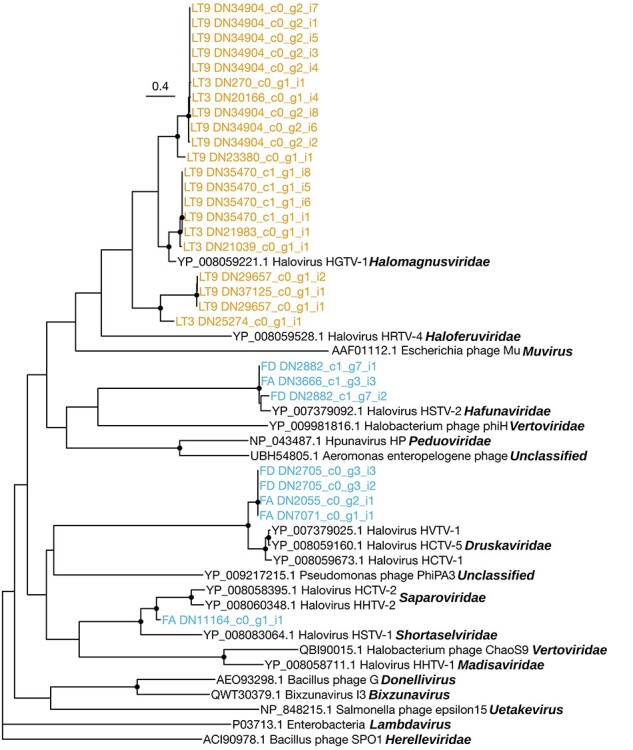
Phylogenetic tree depicting the evolutionary relationships among DNA viruses from the class *Caudoviricetes*, including those identified here. The phylogeny was estimated from an alignment of 400 amino acid residues representing the major capsid protein. Contigs from the Lake Tyrrell libraries are highlighted in orange (top of the tree), while those from the Antarctic lakes are highlighted in blue. Tips labelled in black represent members of the class *Caudoviricetes* and have their family name (or genus where the family is unclassified) at the end of the label in bold italics. Bacillus phage SPO1, a member of the *Herelleviridae*, is used as an outgroup to root this tree. Points on internal nodes denote branches with SH-aLRT ≥80 per cent and bootstrap ≥95 per cent, while the scale bar represents the number of amino acid substitutions per site.

As members of the class *Caudoviricetes* comprised most of the diversity and abundance of DNA viruses in our libraries, we performed a phylogenetic analysis of their major capsid proteins. Contigs representing the major capsid proteins of the class *Caudoviricetes* were translated and aligned with reference capsid proteins from which a phylogenetic analysis was conducted (Fig. 6). This analysis revealed that these contigs fell within the families *Halomagnusviridae, Saparoviridae, Druskaviridae*, and *Hafunaviridae* from the class *Caudoviricetes* (Senčilo and Roine 2014). Specifically, 21 contigs from the Lake Tyrrell libraries were related to Halogeometricum tailed virus 1 (species *Hagravirus HGTV1*), a virus of the *Halomagnusviridae* known to have a myo-like morphology: a long contractile tail and circularly permuted dsDNA genome. The virus contigs sampled from the Antarctic Filla Island libraries fell into three clades. A single contig fell as a sister lineage to Haloarcula californiae tailed virus 2 (species *Samsavirus HCTV2*) and Haloarcula hispanica tailed virus 2 (species *Halohivirus HHTV2*), both of which are members of the *Saparoviridae* and have sipho-like morphology with long non-contractile tails and linear dsDNA genomes. Four contigs grouped in a clade sister to that containing Haloarcula vallismortis tailed virus 1 (species *Tredecimvirus HVTV1*), Haloarcula californiae tailed virus 5 (species *Tredecimvirus HCTV5*), and Haloarcula californiae tailed virus 1 (species *Hacavirus HCTV1*), all of which belong to the *Druskaviridae*. Finally, three contigs were related to Halorubrum sodomense tailed virus 2 (species *Mincapvirus HSTV2*) of the *Hafunaviridae*, and these contigs were ubiquitous and abundant across the Antarctic libraries.

We attempted a similar analysis of the 77 contigs that fell within the family *Pleolipoviridae*. From these, we were able to extract 11 contigs that were most closely related to the major capsid of the *Pleolipoviridae*. However, a BLAST analysis revealed that these were more similar to the genomes of archaeal hosts than to those of archaeal viruses. Specifically, the *Pleolipoviridae*-related contigs had significant hits (average of 81 per cent nucleotide identity) to members of the genera *Saliphagus* and *Natrinema* (both *Natrialbaceae*). As these viruses are known to have members with a prophage stage (Krupovic et al. 2018), it is likely that they represent endogenous viruses precluding meaningful phylogenetic analysis.

## Discussion

We characterised the viromes associated with five hypersaline lakes in Australia and Antarctica. As these lakes are dominated by archaea (Fig. 2; >70 per cent of the microbial population) this provided the opportunity to potentially identify archaeal RNA viruses through meta-transcriptomic sequencing. Samples from Lake Tyrrell in Australia were direct water samples, while those from Antarctic archaea were enriched from stored samples of Antarctic lake water. This is important as cultures, as was the case for our enrichments, often provide an easier way to connect hosts to viruses (Charon, Murray, and Holmes 2021). The opposite is true for environmental samples of microbial communities, although these harbour less viral diversity.

The archaea-enriched Antarctic lake cultures comprised an average of 96 per cent archaea, particularly those from the *Natrialbaceae* and *Halorubraceae* (Fig. 2). Research on the Antarctic Deep Lake community (DeMaere et al. 2013) shows that they are dominated by the *Halorubraceae* species *Hht. litchfieldiae* (isolate tADL), although the *Natrialbaceae* thrived on the conditions of our enrichments and was often a substantial presence in the communities, as also noted by Hamm et al. (2019). The libraries from Lake Tyrrell have communities that are on average 73 per cent archaea (Fig. 2). We captured biomass on 0.1 µm filters from Lake Tyrrell, where an archaea-dominated microbial community had been described (Emerson et al. 2013; Podell et al. 2014). Corroborating previous research, we detail a community composed chiefly of *Hqr. walsbyi*, a member of the *Halobacteriaceae*.

Through the use of a consecutive search protocol we determined that 89 per cent of our contigs were related to previously known sequences (Fig. 1). Further, by utilising virus detection tools, particularly the Palmscan and RdRp-scan approaches that rely on RdRp motif detection and Phyre2 that finds similarity by comparing predicted to database protein structures, we identified 19 candidate virus-like sequences including 12 with similarities to RNA viruses. These, in theory, could be associated with archaea, although this remains unproven and none of these contigs had sequence similarity to archaea CRISPR spacers. Importantly, these candidate contigs were too divergent to place within an evolutionary framework using phylogenetic analyses, and advances in this area will likely require the accurate inference of evolutionary relationships using elements of protein structure. The same was true of the seven putative RdRp sequences identified by LucaProt. Also of note was that none of our candidates represented the phylum *Lenarviricota* that we hypothesised were the most likely group to have archaea-infecting members. It is unclear whether the absence of this group reflects the hypersaline environment, their true absence, or is due to the very high levels of sequence divergence.

Because our libraries are dominated by archaea we suspect that at least some of our virus-like candidates are likely to be associated with archaea, and most had similar dinucleotide representation to reference hypersaline bacteria and archaea. Critically, however, host assignment cannot be proven through metagenomic data alone and additional work is needed in this area. The highest ranking of our virus-like candidates—denoted Deep-1—exhibits similarity to a picornavirus coat protein with high confidence, although this may in part reflect the lack of more divergent structures in the PFAM database. In addition, it has a dinucleotide representation that is most similar to the archaeon *H. litchfieldiae*. Unfortunately, the short length of Deep-1 (459 nt) makes accurate host assignment impossible. The candidates identified through the Palmscan analysis of the Lake Tyrrell libraries (Tyrrell-1 to Tyrrell-13) were greater than 1,200 nt in length. The highest-ranking candidate of these, Tyrrell-1, has a kitrino-like C-motif, although the RdRp motifs in this sequence are not arranged in the usual order. Confirming the candidates as true archaeal viruses will ultimately require isolation and/or imaging of the virus. While it seems likely that the difficulty in identifying RNA viruses in our microbial communities is due to extreme divergence from known viruses, it is possible that the CRISPR-Cas pathway in archaea (Terns and Terns 2011) may also have reduced the level of RNA virus infection.

It is striking how few, if any, RNA viruses were identified in these data compared to those viruses with DNA genomes. Other than the highly divergent sequences described above, we identified no representatives of any known group of RNA viruses, including those from the expansive data set recently generated by Hou et al. (2023) that contained multiple clades of RNA bacteriophage. Although this may in part reflect the use filtration techniques such that any viruses obtained must necessarily be intra-cellular, it is clear that hypersaline environments dominated by archaea represent a hostile environment for RNA viruses. In contrast, we were readily able to detect DNA viruses that were largely members of the tailed viruses of the class *Caudoviricetes* and the family *Pleolipoviridae*. The DNA viruses identified broadly accord with those described previously (Tschitschko et al. 2015), despite this prior study being specific to only Deep Lake in Antarctica. The viruses previously described in Lake Tyrrell (Emerson et al. 2012, 2013) are also from the class *Caudoviricetes* and family *Pleolipoviridae.*

In summary, we have identified candidates for possible RNA viruses in archaea based on analysis of Australian and Antarctic salt lake metagenomes. Although we have not described any definitive archaeal RNA viruses, we are arguably closer to breaking into this elusive RNA virosphere than ever before and the putative viruses identified here clearly merit additional investigation. The barriers to the identification of *bona fide* RNA viruses of archaea remain the lack of tools for archaeal rRNA removal, the lack of characterised archaeal genomes, and the extreme divergence of any potential archaeal RNA virus.

## Supplementary Material

vead057_Supp

## Data Availability

The raw sequencing data generated here are available at the NCBI Sequence Read Archive (SRA) under Bioproject PRJNA916840 with the accession numbers SRR23196177–SRR231961783, SRR24125903, and SRR24125904. Candidate consensus sequences are available on NCBI GenBank with accession numbers OQ889532–OQ889550.
